# *Arf* Induction by Tgfβ Is Influenced by Sp1 and C/ebpβ in Opposing Directions

**DOI:** 10.1371/journal.pone.0070371

**Published:** 2013-08-05

**Authors:** Yanbin Zheng, Caitlin Devitt, Jing Liu, Nida Iqbal, Stephen X. Skapek

**Affiliations:** Division of Hematology-Oncology, Department of Pediatrics, The University of Texas Southwestern Medical Center, Dallas, Texas, United States of America; Indiana University, United States of America

## Abstract

Recent studies show that *Arf*, a *bona fide* tumor suppressor, also plays an essential role during mouse eye development. Tgfβ is required for *Arf* promoter activation in developing mouse eyes, and its capacity to induce *Arf* depends on Smads 2/3 as well as p38 Mapk. Substantial delay between activation of these pathways and increased *Arf* transcription imply that changes in the binding of additional transcription factors help orchestrate changes in *Arf* expression. Focusing on proteins with putative DNA binding elements near the mouse *Arf* transcription start, we now show that Tgfβ induction of this gene correlated with decreased expression and DNA binding of C/ebpβ to the proximal *Arf* promoter. Ectopic expression of C/ebpβ in mouse embryo fibroblasts (MEFs) blocked *Arf* induction by Tgfβ. Although basal levels of *Arf* mRNA were increased by C/ebpβ loss in MEFs and in the developing eye, Tgfβ was still able to increase *Arf*, indicating that derepression was not the sole factor. Chromatin immunoprecipitation (ChIP) assay showed increased Sp1 binding to the *Arf* promotor at 24 and 48 hours after Tgfβ treatment, at which time points *Arf* expression was significantly induced by Tgfβ. Chemical inhibition of Sp1 and its knockdown by RNA interference blocked *Arf* induction by Tgfβ in MEFs. In summary, our results indicate that C/ebpβ and Sp1 are negative and positive *Arf* regulators that are influenced by Tgfβ.

## Introduction

*Arf*, a *bona fide* mammalian tumor suppressor gene transcribed from the *Cdkn2a* locus, encodes p19^Arf^ in an alternative reading frame when compared to p16^Ink4a^, the first gene found at this chromosomal locus [1]. Mouse p19^Arf^ is primarily known to physically interact with and block Mdm2, thereby stabilizing p53 and contributing to cancer surveillance [2]. Genetically engineered mice that lack the first coding exon for *Arf*, but retaining the *Ink4a* coding sequence, develop spontaneous tumors from as early as two months of age [3]. Although *Arf* coding sequence can be deleted in mouse and human tumors, in a substantial number the gene is intact but silenced alone or together with *INK4A* [4], [5]. Therefore, understanding how *Arf* expression is controlled is relevant to understanding a fundamental mechanism that cancer cells utilize to evade its tumor suppressive activity.

A number of findings indicate that transcriptional control of *Arf* is the major determinant of p19^Arf^ protein level and function. Throughout most of the developing mouse embryo, *Arf* expression is essentially silenced [6]. Indeed, our studies reveal that *Arf* expression is detectable only in the developing eye and internal umbilical vessels [7]. Global silencing of its expression is mediated by chromatin remodeling proteins such as Bmi1 since the expression of both *Arf* and *Ink4a* increase when *Bmi1* is deleted in mouse models [8]. In this regard, a long non-coding RNA (*ANRIL*), transcribed anti-parallel to human *ARF* and *INK4a* (and the *INK4b* gene lying further 5′ of *ARF/INK4a*) [9] acts in *cis* to foster CBX7 binding to this region in cultured human PC3 cells [10]. Despite evidence for global repression of the *Cdkn2a* locus, it is also clear that transcription activators contribute to the selective induction or repression of the *Arf* promoter. Examples include E2Fs 1 and 3 [11], [12], [13], [14], Dmp1 [15], [16], AP1 [17], and Pokemon [18]. FoxO proteins are also implicated as *Arf* regulators and they appear to act by binding an element in the first *Arf* intron, far from the transcription start site [19]. It is important to note that many of these conclusions stem from highly tractable cell culture models, but the *in vivo* relevance is less clear in most cases.

Adding to the concept that *Arf* must have tissue-specific control is the fact that the gene plays an essential role in eye development [20]. *Arf*-deficient mice develop persistent hyperplastic primary vitreous (PHPV) that is evident at embryonic day (E) 13.5 and persists in the postnatal period [20]. In this setting, p19^Arf^ blocks the expression of Pdgfrβ, a growth factor receptor that is essential for hyperplastic accumulation of cells in the primary vitreous in the absence of *Arf* [21]. Tgfβ2 is essential for *Arf* expression in the developing mouse [7]; and in cultured MEFs, *Arf* induction by Tgfβ depends on activation of TbrII, Smad 2/3, and p38 Mapk [22]. Interestingly, RNA polymerase II binding to the *Arf* promoter and increased *Arf* mRNA lag substantially behind activation of these pathways and the binding of Smad 2/3 to the *Arf* gene [22]. Moreover, Tgfβ2 has numerous effects during mouse embryo development whereas *Arf* expression is principally localized to the primary vitreous [7]. Both findings indicate that other proteins must cooperate with Smad 2/3 to control *Arf*. Taking advantage of mouse and cell culture-based models, we identify two such cooperating events: de-repression of *Arf* by C/ebpβ down-regulation and loss of promoter binding, and transcriptional activation by Sp1.

## Materials and Methods

All animal studies were reviewed and approved by the Institutional Animal Care and Use Committee at the University of Texas Southwestern Medical Center, Dallas, Texas. Methods such as the use of isoflurane for anesthetization of animals were used to minimize suffering during surgeries.

### Mice, Cells and Reagents

*Arf ^lacZ/+^* [7] mice were maintained in a mixed C57BL/6 × 129/Sv genetic background. *Tgfβ2^+/−^* mice [23] and *C/ebpβ ^+/−^* mice [24], also in a mixed C57BL/6 × 129/Sv genetic backgrounds, were purchased from Jackson Laboratories.

Primary MEFs from wild type (WT), *Arf ^lacZ/lacZ^,* and *C/ebpβ ^−/−^* mice were obtained and cultivated as previously described [6]. MSCV-based retrovirus vectors encoding mouse C/ebpβ [Liver Activating Protein (LAP) isoform] were produced in our laboratory using vectors from Addgene (Cambridge, MA). The following chemical agents were used in some analyses: HLM006474 (HLM), from EMD Millipore Chemicals Inc (Billerica, MA); and Mithramycin A, from Sigma (St. Louis, MO). Tgfβ1 (Tgfβ), obtained from R&D Systems, Inc (Minneapolis, MN), was added to cell culture medium at a dose of 5 ng/ml; an equivalent volume of vehicle (4 mM HCl) was added into the medium as a control.

### Real Time RT PCR

Cell pellets were dissolved in 800 µl Trizol (Invitrogen); total RNA was extracted from Trizol solution after addition of chloroform, precipitated with isopropanol, and dissolved in water. Two µg total RNA was use to synthesize cDNA with Superscript III RT kits (Invitrogen) according to the manufacturer’s recommendations. Then, quantitative RT-PCR (qRT-PCR) was performed using Fast SYBR Green Master mix and a model 7900 HT Fast Cycler instrument (both from Applied Biosystems). The primers were as follows: *Arf*: 5′-TTCTTGGTGAAGTTCGTGCGATCC-3′ (forward) and 5′-CGTGAACGTTGCCCATCAT CATCA-3′ (reverse); *C/ebpβ*: 5′-GTTTCGGGACTTGATGCAAT-3′ (forward) and 5′- CCCCGCAGGAACATCTTTA-3′ (reverse); *Sp1:* 5′-TCATGGATCTGGTGGTGATGGG-3′ (forward) and 5′-GCTCTTCCCTCACTGTCTTTGC-3′ (reverse); *Gapdh*: 5′-TCAACAGCAACTCCCACTCTTCCA-3′ (forward) and 5′-ACCCTGTTGCTGTAGCCGTAT TCA-3′ (reverse). Results are pooled from three separate experiments.

### Western Blotting and β-Gal Assay

Cells were collected, lysed, separated by SDS-PAGE and transferred to PVDF membrane with 50–100 µg total protein per sample. The membrane was incubated with primary antibody for two hours, washed trice in Tris-Buffered Saline Tween-20 (TBST) for 15 minutes each time; and then incubated with horseradish peroxidase (HRP)-labeled secondary antibody for one hour. After washing in TBST, the membrane was incubated with 2 ml ECL (GE Healthcare Life Sciences) for 5 minutes and visualized by exposure to film. β-galactosidase assays were performed in *Arf ^lacZ/lacZ^* MEFs as previously described [7] using a commercial kit (Applied Biosystems; Foster City, CA). For western blotting, antibodies directed against the following proteins were utilized: C/ebpβ, and Hsc70 (Santa Cruz Biotechnology, Inc; Santa Cruz, CA); phospho-p38 Mapk, and phospho-Smad2 (Cell Signaling Technology; Danvers, MA); and p19^Arf^ (Abcam Inc; Cambridge, MA). Experimental findings were confirmed in at least two independent experiments, with quantitative data from β-galactosidase assays pooled from all representative experiments.

### Laser Capture Microdissection (LCM)

LCM was done as previously described [25]. Briefly, mouse embryos ware harvested at E13.5 for LCM. Embryo heads were immediately embedded in OCT freezing medium without fixation. Fourteen µm thick sections were cut on a CryoStar NX70 cryostat, which were mounted on PEN Membrane Metal Slides (Applied Biosystems) and stained with hematoxylin and eosin (H&E) (Molecular Machines & Industries AG; Glattbrugg, Switzerland). LCM was carried out using an Arcturus Veritas Microdissection System. Cells in the vitreous, lens, and retina were dissected from each eye and collected separately. Samples were pooled from at least 5 microdissected sections from the same embryo. Total RNA was extracted using an Arcturus PicoPure LCM RNA isolation kit (Applied Biosystems) and the expression of specific genes was analyzed with real time RT-PCR as described above.

### ChIP Assay

Chromatin immunoprecipitation (ChIP) experiments were performed as previously described [22]. Briefly, wild type MEFs (3×10^6^/ChIP) were treated with Tgfβ (5 ng/ml) or vehicle for 1.5, 24 or 48 hours. Cells were cross-linked and sonicated, and then subjected to immunoprecipitation using antibodies against C/ebpβ (sc150, Santa Cruz Biotechnology, Inc., Santa Cruz, CA), or Sp1 (sc59, Santa Cruz). Rabbit IgG (sc2027, Santa Cruz) was used as a negative control. Protein A/G sepharose beads (sc2003, Santa Cruz) were used to collect the antibody-chromatin complexes. The beads were washed sequentially with low salt, high salt, LiCl and TE buffers (Upstate ChIP Kit, Millipore) and eluted in 0.1 M NaHCO3, 1% SDS. Cross-linking was reversed by incubation at 67°C overnight, and the genomic DNA was extracted using Qiagen PCR Purification Kit. Quantitative analysis of the precipitated and input DNA was carried out using specific primer sets and Fast SYBR green master mix on a model 7900 HT Fast Cycler instrument (both from Applied Biosystems). The primer sets for proximal promoter regions of *Arf* were as follows: 5′-AGATGGGCGTGGAGCAAAGAT-3′ (forward) and 5′- ACTGTGACAAGCGAGGTGAGAA (reverse).

### siRNA

We purchased siRNA against mouse *SP1* (catalog # 74195; Life Technologies, Grand Island, NY). The siRNA was dissolved in 1× siRNA buffer (Dharmacon) and used for transfection (100 nM final concentration). Scrambled siRNA (siGENOME Non-Targeting siRNA #3, Dharmacon) was used as control. 24 hours after the initial transfection, the cells were treated with either Tgfβ or vehicle, and they were harvested 48 hours later for western blotting or RT-PCR.

### Statistical Analysis

Quantitative data are presented as the mean±S.D. from three or more representative experiments. Statistical significance (p value <0.05) was calculated using Student’s t test.

## Results

**Figure 1:**
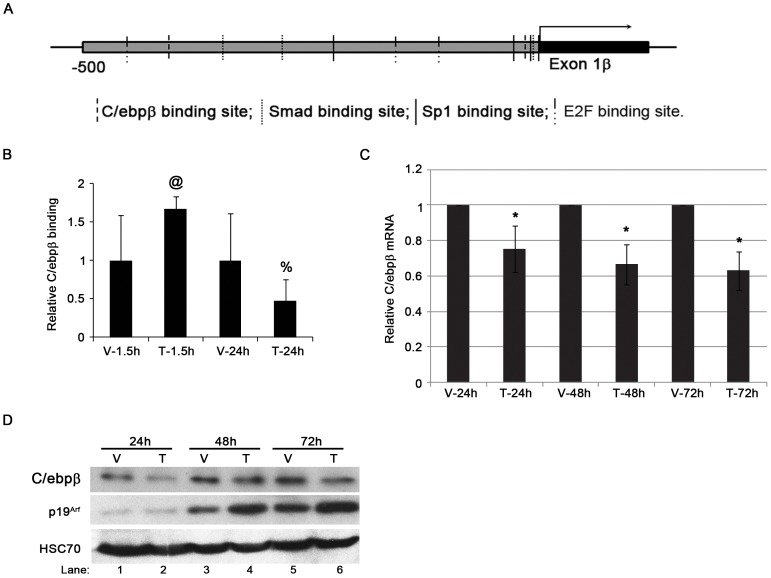
Inverse correlation of C/ebpβ and *Arf* expression during Tgfβ treatment. (A). Schematic diagram showing potential C/ebpβ, Smad, Sp1 and E2F binding sites at the *Arf* promoter. (B). Tgfβ decreases C/ebpβ binding to the *Arf* locus in MEFs. Quantitative analysis of representative chromatin immunoprecipitation (ChIP) assays of using wild type MEFs exposed to vehicle (V) or Tgfβ (T) for 1.5 hours or 24 hours. ChIP assay was carried out using antibodies specific to C/ebpβ and IgG. Immunoprecipitated DNA and input DNA were amplified with primers for proximal regions genomic *Arf* promoter. *p*-values as follows: 0.1 (@) and 0.2 (%) for Tgfβ versus corresponding vehicle. (C). Quantitative analysis of real time, RT-PCR using total RNA isolated from WT MEFs shows the expression of C/ebpβ mRNA changes during Tgfβ treatment up to 72 hours. The data is plotted as the fold changes of target genes from cells treated with Tgfβ (T) (5 ng/ml) versus the same cells treated with vehicle (V) (4 mM HCl). The significant changes between Tgfβ treatment and vehicle treatment was marked as * (p<0.05). (D) Representative western blot of lysates from wild type MEFs treated with Tgfβ (T) and vehicle (V) at different time points showing the inverse correlation of C/ebpβ and *Arf* protein expression.

Recognizing the substantial delay between Smad binding to the *Arf* promoter and increased synthesis of *Arf* primary transcript [22], we considered potential roles for other transcription factors whose function might be influenced by Tgfβ. Among those, C/ebpβ was an attractive candidate because previous work had implicated it as an *Arf* repressor in primary epidermal keratinocytes [26], and putative consensus DNA binding elements are found within 500 bp 5′ to the *Arf* translation initiation codon (Figure 1A). Utilizing chromatin immunoprecipitation (ChIP), we demonstrated that C/ebpβ was bound to this region in cultured mouse embryo fibroblasts (MEFs) at passage 3 (YZ and SXS, unpublished data).

**Figure 2:**
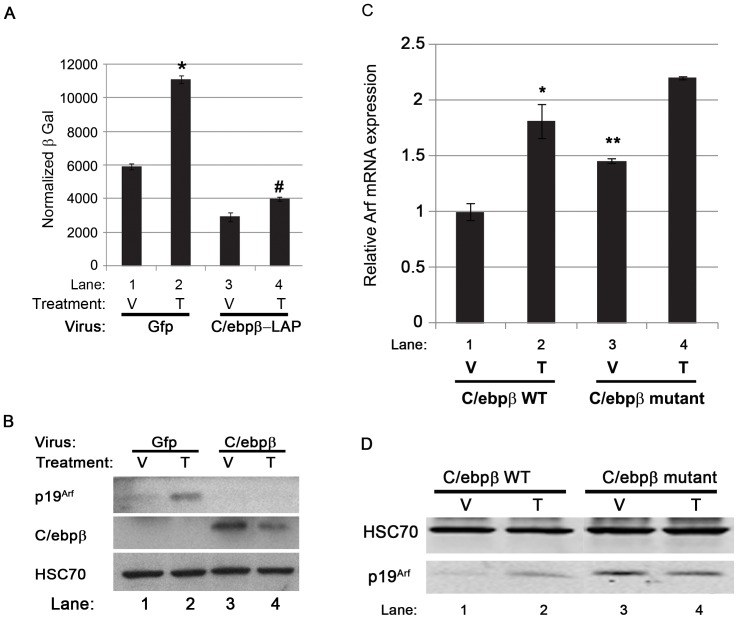
The effects of overexpression or absence of C/ebpβ on *Arf* induction by Tgfβ. (A). β-galactosidase activity in *Arf ^lacZ/lacZ^* MEFs showing the effects of ectopically-expressed C/ebpβ (LAP form) on *Arf* induction following 48 hour exposure to Tgfβ. Significant increase (*) and decrease (#) of Arf^lacZ^ expression is represented in the figure. *, #, p<0.05. (B) Representative western blot for the indicated proteins using lysates from wild type MEFs, exposed to 48 hours of Tgfβ (T) and vehicle (V) after transduction using Gfp- or C/ebpβ (LAP form)-expressing retrovirus. (C) qRT-PCR using total RNA isolated from *C/ebpβ ^+/+^* and *C/ebpβ ^−/−^* MEFs exposed to vehicle (V) or Tgfβ (T) for 48 hours. Differences in transcript level between Tgfβ- and vehicle-treated *C/ebpβ ^+/+^* MEFs are significant [p<0.05 (*)]. Differences in transcript level between vehicle-treated *C/ebpβ ^+/+^* and *C/ebpβ ^−/−^* MEFs are significant, too [p<0.05 (**)]. (D) Representative western blot for the indicated proteins using lysates from *C/ebpβ ^+/+^* and *C/ebpβ ^−/−^* MEFs exposed to vehicle (V) or Tgfβ (T) for 48 hours.

We next investigated whether Tgfβ influenced the binding of endogenous C/ebpβ to the *Arf* promoter. We previously established that Smad 2/3 binding to elements in the proximal *Arf* promoter (Figure 1A) is enhanced within 1.5 hours following the addition of Tgfβ2 to the culture medium, whereas RNA polymerase II (RPolII) binding is not increased until 24 hours, after which *Arf* mRNA increases [22]. Paralleling the delayed RPolII binding, C/ebpβ localization to a proximal promoter element in the *Arf* promoter was diminished at 24 hours following an initial increase at 1.5 hours (Figure 1B). Interestingly, Tgfβ stimulation diminished *C/ebpβ* mRNA and protein between 24 and 72 hours (Figures 1C and D). The effect on C/ebpβ protein expression was evident when it was ectopically expressed (Figures 2B, lane 3 versus 4), implying that the decreased repression was not simply due to decreased transcription of the native mRNA. Of note, the fact that p19^Arf^ level did not strictly inversely correlate with C/ebpβ (Figure 1D, lane 3 versus 1) indicates that other factors, such as cell “culture shock” that has been described for cultured mouse fibroblasts [27], must play a role in expression of this tumor suppressor and these other factors maybe be independent of Tgfβ signaling (see more below).

We confirmed that ectopically expressed C/ebpβ blunted *Arf* transcription by showing that β-galactosidase activity was repressed in cultured *Arf ^lacZ/lacZ^* MEFs infected with retrovirus encoding the liver-enriched activator protein (LAP) isoform of C/ebpβ, which includes a transactivation domain [28], [29] (Figure 2A, lane 3 versus 1). Consistent with the concept that p19^Arf^ expression is primarily controlled by *Arf* transcription, Western blotting showed that ectopic C/ebpβ also diminished the low basal p19^Arf^ evident in wild type MEFs at passage 3 (Figure 2B, lane 3 versus 1). Further, ectopic expression of C/ebpβ also blunted Tgfβ-dependent induction of *Arf* transcription and p19^Arf^ expression in cultured MEFs (Figures 2A and B, lane 2 versus 4). These data indicate that C/ebpβ can repress *Arf* expression in MEFs in a manner that is dominant over Tgfβ-dependent induction of p19^Arf^.

We next took advantage of *C/ebpβ ^−/−^* mice to begin to address whether de-repression by C/ebpβ down-regulation contributes to *Arf* induction by Tgfβ. *C/ebpβ ^−/−^* mice have been previously shown to exhibit increased postnatal lethality, abnormal hematopoiesis, abnormal glucose homeostasis and immune system defects, among their abnormalities [24], [30]. The mice were generated by introducing a MCI-Neo poly(A)+ mutation at the 3′ terminus of *C/ebpβ* to abolish translation of the LAP and LIP isoforms [24]. As previously described [26], analysis of cultured MEFs derived from wild type and *C/ebpβ ^−/−^* embryos demonstrated that basal *Arf* mRNA and p19^Arf^ protein were increased upon *C/ebpβ* loss (Figure 2C and D, lane 3 versus 1). Despite the increased baseline *Arf* expression, though, absence of C/ebpβ only minimally influenced the further induction of *Arf* mRNA by Tgfβ (Figure 2C, compare lane 4 versus 3 with 2 versus 1). This further increase in p19^Arf^ was not as evident by western blotting (Figure 2D, compare lane 4 versus 3 with 2 versus 1), suggesting that additional factors may act by post-transcriptional mechanisms to control p19^Arf^ protein level. Taken together, these findings indicate that loss of *C/ebpβ* binding to the *Arf* promoter cannot fully account for the increased *Arf* mRNA in response to Tgfβ stimulation.

**Figure 3:**
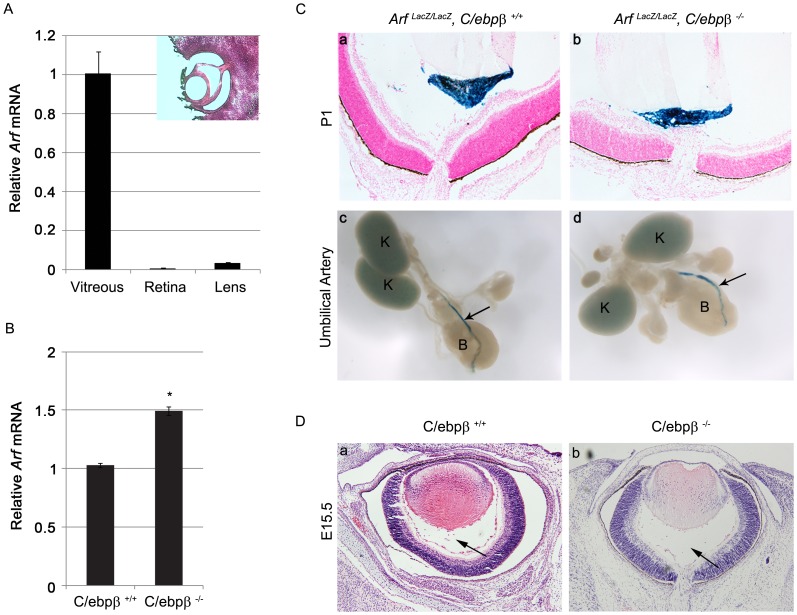
Loss of C/ebpβ increases *Arf* mRNA expression in vitreous of developing eye. (A). qRT-PCR analysis using total RNA isolated from the vitreous (V), lens (L) and retina (R) from E13.5 WT mouse embryos. Expression was normalized to that of *Gapdh*. (B) qRT-PCR analysis using total RNA isolated from the vitreous from E13.5 *C/ebpβ ^+/+^* and *C/ebpβ ^−/−^* mouse embryos. Expression was normalized to that of *Gapdh*. (C) *Arf* expression is limited to previously identified sites in *C/ebpβ ^−/−^* mice during development. (a, b) Representative photomicrographs of hematoxylin- and eosin-stained and X-Gal stained slides of P1 mouse eye of the indicated genotype. Note that *Arf*-expressing cells are limited to the vitreous (blue staining) in the *Arf ^lacZ/lacZ^, C/ebpβ ^−/−^* embryo, similar to the littermate *Arf ^lacZ/lacZ^, C/ebpβ ^+/+^* control embryo. (c,d) Representative whole-mount, E13.5 embryo from mice of the indicated genotype, following X-gal staining. Note that *Arf*-expressing cells are limited to the umbilical artery (arrow) in the *Arf ^lacZ/lacZ^, C/ebpβ ^−/−^* embryo, similar to its littermate *Arf ^lacZ/lacZ^, C/ebpβ ^+/+^* control embryo. K, kidney; B, bladder. (D). Representative photomicrographs of hematoxylin- and eosin-stained slides of E15.5 embryos showing there is no primary vitreous hyperplasia in *C/ebpβ ^−/−^* embryos. Arrows denote the cellular area of the primary vitreous.

We extended our studies to the *in vivo* setting by examining how the presence or absence of C/ebpβ influences *Arf* expression and Tgfβ2 effects in the developing vitreous, the only well-characterized site of p19^Arf^ activity in the developing mouse embryo [7], [21]. At E13.5, *Arf* mRNA is principally detected in the primary vitreous (Figure 3A), where p19^Arf^ represses Pdgfrβ expression to block vascular mural cell hyperplasia [21], [25]. Consistent with its role as a *bona fide* repressor, *Arf* mRNA was elevated in the primary vitreous of *C/ebpβ ^−/−^* embryos as compared to wild type (Figure 3B). In addition to de-repressing *Arf* expression in a tissue known to express the transcript, we investigated whether loss of C/ebpβ was sufficient to drive ectopic *Arf* expression beyond its normal expression pattern. Utilizing *Arf ^lacZ/lacZ^* animals in which the β-galactosidase reporter reflects *Arf* mRNA [7], we did not find enhanced *Arf* expression in ocular tissues that do not normally express *Arf*, nor did its expression in genitourinary structures extend beyond the internal umbilical artery (Figure 3C). Finally, we found no apparent ocular abnormalities at E15.5 or in the postnatal period (Figure 3D and additional data not shown), indicating that the increased *Arf* mRNA was not obviously detrimental.

**Figure 4:**
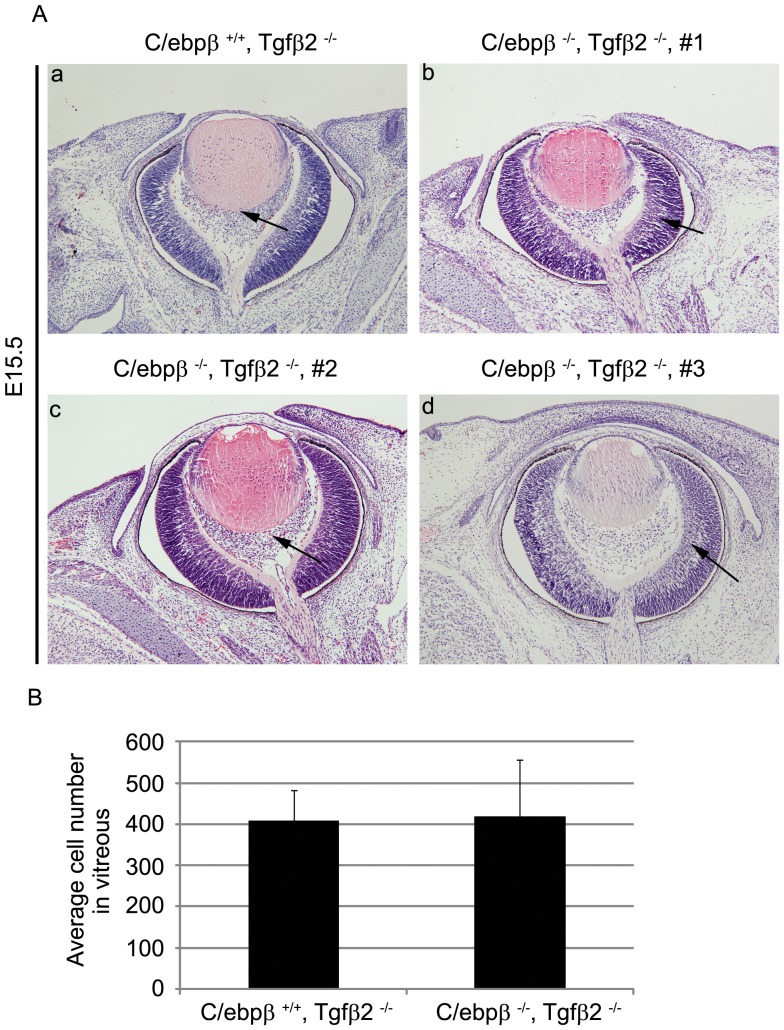
Loss of C/ebpβ is insufficient to rescue PHPV like eye phenotype of Tgfβ2 KO mouse. (A) Representative photomicrographs of hematoxylin- and eosin-stained slides of E15.5 embryos showing the primary vitreous hyperplasia in *C/ebpβ^+/+^, Tgfβ2^−/−^* embryos (a) is NOT corrected by additional loss of expression of C/ebpβ in *C/ebpβ ^−/−^, Tgfβ2^−/−^* embryos (b-d). Arrows denote the cellular area of the primary vitreous. (B) Quantitative analyses show that the average cell numbers in the vitreous have little change in *C/ebpβ ^−/−^, Tgfβ2^−/−^* embryos at E13.5 as compared with *C/ebpβ ^+/+^, Tgfβ2^−/−^* littermates.

We previously established that p19^Arf^ expression is diminished in the primary vitreous of *Tgfβ2^−/−^* embryo eyes and this results in primary vitreous hyperplasia, mimicking that observed in *Arf ^−/−^* embryos [7]. That exogenous Tgfβ1 reverses this phenotype in *Tgfβ2^−/−^* embryos – but not in *Arf ^−/−^* embryos – demonstrates that p19^Arf^ is the key Tgfβ-dependent target that prevents primary vitreous hyperplasia [22]. If Tgfβ2 solely acts to reverse C/ebpβ-driven *Arf* repression, the primary vitreous hyperplasia in *Tgfβ2^−/−^* embryos should be rescued in *C/ebpβ ^−/−^* embryos. We investigated this by analyzing the ocular phenotype in *Tgfβ2^−/−^* embryos that had or lacked *C/ebpβ*. Our analyses demonstrated that the eyes of *Tgfβ2^−/−^* embryos were indistinguishable from those lacking both genes (Figure 4A and B). That the absence of an *Arf* repressor cannot reverse the developmental abnormality illustrates that Tgfβ2 likely also influences a positively acting factor to drive p19^Arf^ expression in the primary vitreous.

Considering potential positive regulators of *Arf*, E2Fs and Sp1 are reasonable candidates based, in part, on DNA binding elements near the *Arf* transcription start site (Figure 1A). E2Fs have been proven to participate in *Arf* regulation in various cell contexts [11], [14], [31], [32]. Sp1 has been implied to be important in *Arf* regulation because deletion of potential Sp1 binding sites diminishes *Arf* promoter expression, and because Sp1 can bind to the *Arf* promoter [11], [33].

**Figure 5:**
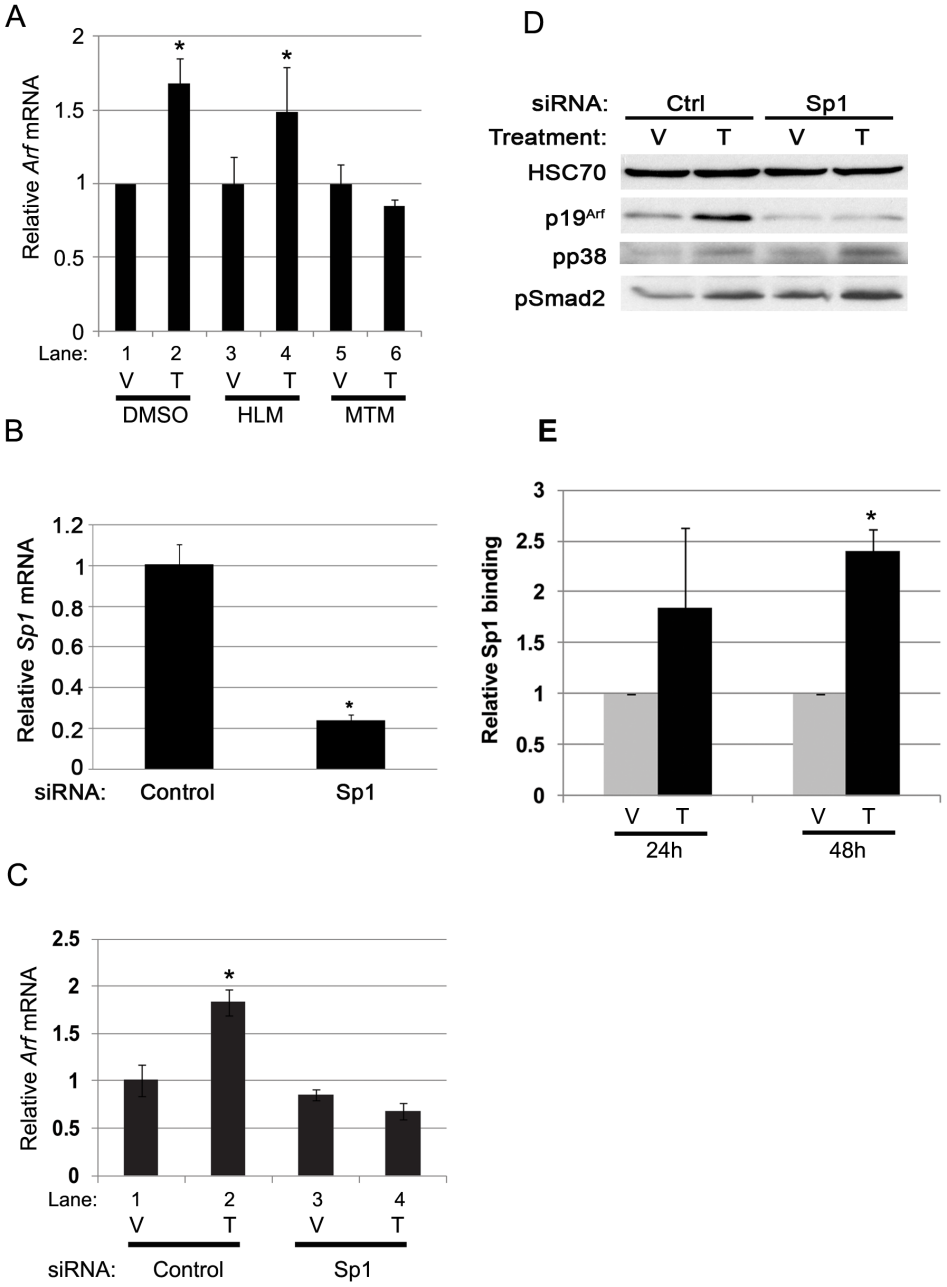
Inhibition or knockdown of Sp1 blocks *Arf* mRNA induced by Tgfβ. (A) qRT-PCR analysis using total RNA isolated from WT MEFs treated with Sp1 inhibitor, mithramycin A (MTM), E2F inhibitor, HLM006474 (HLM) and control DMSO, following 48 hour exposure to Tgfβ (T) or vehicle (V). The significant changes between Tgfβ treatment and vehicle treatment is marked as * (p<0.05). (B) qRT-PCR analysis of Sp1 using total RNA isolated from WT MEFs treated with either siRNA control (Scram), or siRNA targeting mouse Sp1 as indicated for 48 hours. *, p<0.05. (C) qRT-PCR analysis using total RNA isolated from WT MEFs treated with Tgfβ (T) or vehicle (V) for 48 hours following 24 hours transfection with either siRNA control (Scram), or siRNA targeting mouse Sp1 as indicated. Sp1 knockdown significantly dampens the induction of *Arf* mRNA by Tgfβ (*, p<0.05). (D) Representative western blot for the indicated proteins using lysates from wild type MEFs treated with Tgfβ (T) or vehicle (V) for 48 hours following 24 hours transfection with either siRNA control (Scram), or siRNA targeting mouse Sp1 as indicated. (E) Tgfβ promotes Sp1 binding to the *Arf* locus in MEFs. Quantitative analysis of representative ChIP assays using wild type MEFs exposed to vehicle (V) or Tgfβ (T) for 24 hours or 48 hours. ChIP assay was carried out using antibodies specific to Sp1 and IgG as control. Immunoprecipitated DNA and input DNA were amplified with primers for proximal region of *Arf* promoter. *, p<0.05 for Tgfβ versus corresponding vehicle.

To begin to test whether these candidates act in response to Tgfβ, we first investigated whether chemical inhibition of either pathway interfered with *Arf* induction by Tgfβ. We utilized HLM006474 (HLM), which inhibits the DNA-binding activity of E2Fs [34], and mithramycin A (MTM) which, among other things, interferes with Sp1 binding to GC-rich DNA [35]. Induction of *Arf* mRNA by Tgfβ proceeded unabated in the absence or presence of HLM (Figure 5A, lane 3 and 4 versus lane 1 and 2), even though it restored the repression of other E2F-dependent genes like PAI-1 [36](YZ and SXS, unpublished data). In contrast, MTM blocked *Arf* mRNA induction (Figure 5A, land 5 and 6 versus lane 1 and 2), but MTM did not significantly block Smad 2/3 binding to the proximal region of *Arf* promoter (YZ and SXS, negative data not shown). To exclude potential off-target effects of MTM, we showed that transient *Sp1* knockdown by siRNA transfection (Figure 5B) also blocked *Arf* mRNA and protein induction by Tgfβ (Figures 5C and D). Of note, *Sp1* knockdown did not block phosphorylation of Smad 2/3 or p38 Mapk (Figure 5D), two events that are required downstream of Tgfβ2 [22]. Finally, ChIP demonstrated that the minimal Sp1 binding to the proximal *Arf* promoter at baseline was significantly increased by Tgfβ at 24 and 48 hours (Figure 5E and additional data not shown), paralleling the time course for *Arf* mRNA increase we previously described [22]. These findings suggest that direct binding of Sp1 to the *Arf* promoter is required for Tgfβ to augment p19^Arf^ expression.

## Discussion

We recently demonstrated that Tgfβ is an essential regulator of *Arf* during eye development [7], [22]. However, *Arf* expression is limited given the protean effects of Tgfβs during mouse embryo development [7], and *Arf* mRNA induction is delayed following immediate Smad 2/3 binding to the promoter [22]. Both suggest that *Arf* expression is orchestrated by Tgfβ-dependent changes in transcriptional regulators beyond the Smad proteins. Our new data indicate that Sp1 and C/ebpβ represent such cooperating factors, influencing *Arf* induction in opposing ways. We have the following evidence: First, ectopic expression of C/ebpβ blocked *Arf* induction by Tgfβ. Second, C/ebpβ binding to the *Arf* promoter is diminished by Tgfβ treatment in a time frame coincident with *Arf* mRNA induction. The concept that Tgfβ orchestrates de-repression of *Arf* by C/ebpβ down-regulation *in vivo* is supported by the fact that *Arf* expression in the vitreous is elevated in *C/ebpβ ^−/−^* animals. However, absent the essential *Arf* inducer – Tgfβ2– loss of C/ebpβ is not sufficient to correct the PHPV-like eye phenotype in *Tgfβ2^−/−^* mice; hence, removing C/ebpβ repression is not the whole story. Searching for a positive trans-acting factor induced by Tgfβ, we found chemical and genetic evidence supporting a role for Sp1. In summary, our data provide new insight into the molecular basis underlying *Arf* control by Tgfβ during eye development, and this may inform our understanding of certain disease processes.

Our work extends previous reports implicating both C/ebpβ and Sp1 as potential regulators of p19^Arf^ expression. That C/ebpβ can repress *Arf* was previously suggested primarily by the elevated *Arf* mRNA and protein observed in *C/ebpβ ^−/−^* keratinocytes in culture and in the adult mouse [26]. Sp1 is well known to bind to GC-rich promoter elements [37], [38], and the mouse and human *Arf* promoters contain numerous Sp1 binding sites within CpG islands [15], [33]. Several previous studies showed the potential importance of Sp1 binding to the human *ARF* promoter in cultured cells [11], [39]. However, the potential physiological importance of either in *Arf* regulation is not yet clear. For example, *C/ebpβ ^−/−^* mice are completely refractory to chemically induced skin cancer [40], which concept is consistent with higher p19^Arf^ expression as a tumor suppressor. However, *Arf* does not seem to play a role in tumor resistance in this model [26]. Nonetheless, our findings demonstrating increased *Arf* mRNA in the vitreous of *C/ebpβ ^−/−^* embryos indicates that C/ebpβ can repress *Arf* in a normal developmental context. The lack of widespread *Arf* promoter activation in these embryos and newborn *Arf ^lacZ/lacZ^, C/ebpβ ^−/−^* mice, though, still highlights the importance of tissue-specific positive transcriptional regulators of *Arf*.

The fact that the phenotype due to blunted *Arf* expression in Tgfβ2-deficent embryo eyes was not reversed in animals also lacking *C/ebpβ* provides additional *in vivo* evidence for the importance of positively-acting factors. That Sp1 cooperates with Smad signaling is consistent with previous findings that Tgfβ2 regulates p15^Ink4b^ through direct Sp1 binding to the promoter [41], [42]. Sp1 also collaborates with Smad proteins to induce the expression of vimentin in cultured cells undergoing the epithelial-mesenchymal transition in response to Tgfβ [43]. Our preliminary work shows that decreased expression of C/ebpβ in response to Tgfβ depends on TbrII and Smad 2/3 activation (YZ and SXS, unpublished data), but we do not yet know whether Sp1 binding to the *Arf* promoter similarly depends on the activation of that pathway. Sp1 is also known to work cooperatively with E2Fs [44], which are also implicated as both positive and negative regulators of *Arf* [11], [31], [32], [45]. Our finding that HLM does not significantly block *Arf* induction by Tgfβ suggests that Sp1 seems to act independently of E2Fs in this context. It will obviously be important to demonstrate the functional importance of Sp1 *in vivo* using our mouse model for PHPV. Regrettably, *Sp1^−/−^* mice display an embryonic lethal phenotype at E11.5, before primary vitreous development [46]. Tissue specific *Sp1* knockout using a Wnt1-Cre driver would be very informative.

Finally, we have carried out this line of investigation in the mouse to gain insight into human diseases, like cancer and PHPV. Repression of human *ARF* expression is a relatively common mechanism by which cancers can evade this tumor suppressor activity [47]; presumably, restoring *ARF* expression could represent a novel therapeutic approach, especially for that subset of cancers also retaining wild type p53. As a human disease, PHPV is typically sporadic, but several reports of familial disease suggest that it could have an underlying genetic basis [48], [49], [50]. C/ebpβ is frequently expressed in human cancer and has been implicated as an oncogenic factor (as in the keratinocyte model noted above) [26], [40] or tumor suppressor with the capacity to foster senescence [51], [52]. These disparate effects may be due, in part, to the capacity of C/ebpβ to form homo- and heterodimeric complexes with either activating or transcriptional repressive activity [28]. Sp1, too, could act as a Tgfβ-dependent tumor suppressor, by controlling *Ink4b* [41], [42] or *Arf* (this work), or as an oncogene by facilitating EMT [43]. Again, one could envision that the net effect of Sp1 could depend on the underlying cellular or genetic context. As more sophisticated, “next-generation” genome sequencing and analytical tools are applied – particularly to diseases like PHPV – the role for these genes might be revealed.
